# Microtubule teardrop patterns

**DOI:** 10.1038/srep09581

**Published:** 2015-03-31

**Authors:** Kosuke Okeyoshi, Ryuzo Kawamura, Ryo Yoshida, Yoshihito Osada

**Affiliations:** 1 RIKEN Advanced Science Institute, 2-1 Hirosawa, Wako-shi, Saitama 351-0198, Japan; 2 Department of Materials Engineering, Graduate School of Engineering, The University of Tokyo, 7-3-1 Hongo, Bunkyo-ku, Tokyo 113-8656, Japan

## Abstract

Several strategies for controlling microtubule patterns are developed because of the rigidity determined from the molecular structure and the geometrical structure. In contrast to the patterns in co-operation with motor proteins or associated proteins, microtubules have a huge potential for patterns via their intrinsic flexural rigidity. We discover that a microtubule teardrop pattern emerges via self-assembly under hydrodynamic flow from the parallel bundles without motor proteins. In the growth process, the bundles ultimately bend according to the critical bending curvature. Such protein pattern formation utilizing the intrinsic flexural rigidity will provide broad understandings of self-assembly of rigid rods, not only in biomolecules, but also in supramolecules.

Microtubules, a form of cytoskeletal protein, are tubular assemblies of α/β-tubulin heterodimer molecules that form rigid cylindrical filaments with diameters of 25 nm and lengths of tens of micrometers. Microtubules play vital roles in assisting mechanical events of a cell such as cell division and mass transportation - processes that are observed on up to the order of micrometers to tens of micrometers, depending on the cell size. Furthermore, microtubules have significant potential for showing flagellar swimming motility, cilia arrays wave propagation, and plant cell wall patterns over cell scales, on the order of tens to hundreds of micrometer length. In contrast, *in vitro* achieves unique motions and patterns with bending such as the ring-shaped assembly of microtubules, the dynamic vortex pattern, or active microtubule networks driven by integration with motor proteins^1^,^2^,^3^,^4^.

Because of their geometrically determined structure and flexural rigidity, the bending properties of microtubule bundles have been widely researched from the perspective of materials science^5^,^6^,^7^,^8^,^9^. Furthermore, microtubule bundles on the large-scale tend to exhibit bending flexibility, structural stability for parallel orientation, and co-operability in forming patterns^10^,^11^,^12^,^13^,^14^,^15^,^16^. Actually, a flagellum, composed of a microtubule bundle and motor proteins succeeds in maintaining oscillation by using a periodic motion^17^,^18^. The fine motion is achieved by converting chemical energy, e.g., ATP into mechanical energy on a parallel microtubule bundle, which generates hydrodynamic flow. Focusing on the plastic deformation, the shape transformation of microtubule bundle under hydrodynamic flow is expected to be useful for constructing a well-oriented structure *in vitro*.

Here we introduce remarkable phenomena - that microtubule bundles are capable of bending flexibly under hydrodynamic flow to form teardrop pattern. This growth process precisely represents that straight rigid rods with a high-aspect ratio in parallel orientation are converted into a macroscopically-nonlinear structure with pattern formation. Differing from the dynamic behavior of microtubule bundles, driven by chemical energy with motor proteins in other published works, this teardrop patterns is driven by hydrodynamic flow without any other proteins. The bundles in parallel orientation are prepared on a gas-liquid interface by using the interfacial tension. A load of hydrodynamic flow is applied to the condensed microtubule bundles, causing accumulation of the streamed-line structure to bend ultimately, and also to break according to the critical curvature of microtubules. The growth mechanism, from the bundles to the teardrop pattern as a higher structure, is verified (Fig. 1A).

## Results and Discussion

Microtubule samples for fluorescence microscopic observation were prepared between two glass slides under constant temperature (~25°C) and simply-conditioned atmosphere. A thin liquid layer of microtubule (~1.5 μL: 18 mm × 18 mm × ~5 μm) was prepared by placing a coverglass on a sample droplet on a slide glass (Fig. S1A). Just after a coverglass was placed on a sample droplet, the solution roughly formed a cylindrical shape with circular base of 2–3 mm diameter (Fig. S1B (*B_1_, S_1_*)). When the coverglass is subsequently lowered for spreading out the solution, the area of base reaches 18 × 18 mm^2^ (Fig. S1B (*B_3_, S_3_*)). During this process, the shape of the sample solution would change according to the following equation:
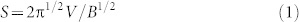


Here, geometries of the cylinder: *V = πr*^*2*^*h, S = 2πrh, B = πr*^*2*^. *V*: volume of the solution (constant), *S*: area of side, *B*: area of base, *r*: radius, *h*: height. Referring to this equation, the initial change in the area of the side from *state 1 (B_1_, S_1_)* to *state 2 (B_2_, S_2_)* in Fig. S1B is calculated, *S_2_/S_1_* = 1/9 ~ 1/6. This means that the area of the gas-liquid interface decreases significantly by lowering the coverglass. Simultaneously in the process of the height lowering, a bending limitation generates in the Z-direction, which induces the transformation in the XY-plane direction (Fig. S2).

**Figure 1:**
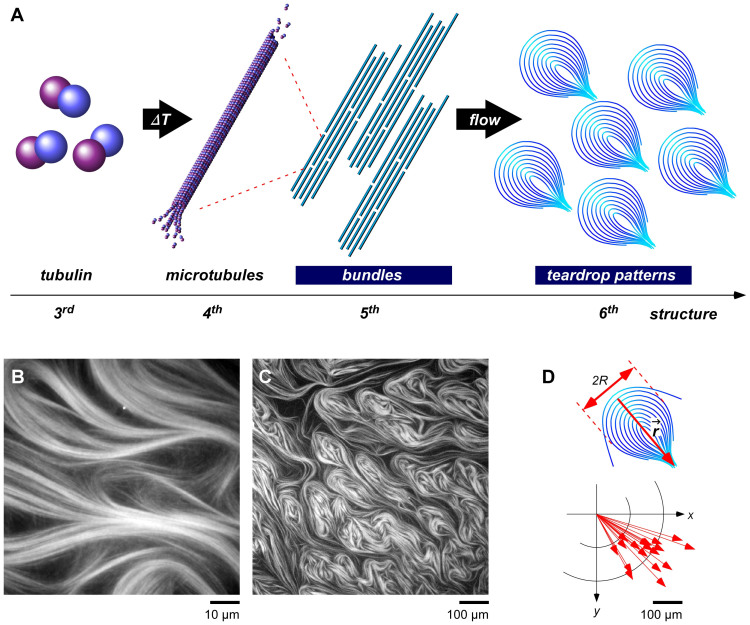
Microtubule teardrop patterns with hierarchy. (A), Hydrodynamic-flow-induced hierarchization of microtubule bundles toward teardrop patterns. (B), (C), Fluorescence microscopy images of microtubules in thin layer at different scales. (D), Microtubule teardrop model for image C. 2*R*: width of teardrop; *r*: vector from arc center to tail of teardrop.

Just after the process from *state 1* (*h_1_* = ~500 μm) to *state 2* (*h_2_* = ~5 μm), parallel bundles were observed along the gas-liquid interface (Fig. S1C). Besides, following bundles simultaneously flow from the center to the outside of the thin layer (Fig. S1D). The flowing bundles were monitored, as shown in Movie S1. The flow of the microtubule bundles could be easily observed in the direction perpendicular to the long axis of the microtubules. The microtubules apparently locate alongside the gas-liquid interface at initial state, and form bundles because of interfacial tension immediately after a coverglass is placed on a sample droplet (*state 1*, Fig. S1A). The microtubules parallel orientation was also confirmed by cross-polars light observation (Fig. S3). The hydrodynamic flow stopped within several tens of minutes after setting the samples between flat glass slides. As shown in Fig. 1B, the microtubule bundles plastically bent while remaining in bundles^19^. The curved bundles formed a condensed pattern homogeneously over a large area on the order of millimeters. The curved bundles were ~100 μm wide (2*R*) in outer diameter and more than 200 μm long from top to tail and were teardrop shaped (Fig. 1C). The patterns were stably maintained for more than one day. Furthermore, the center of the arc to the tail of the teardrop pattern was oriented in the same direction (Fig. 1D).

**Figure 2:**
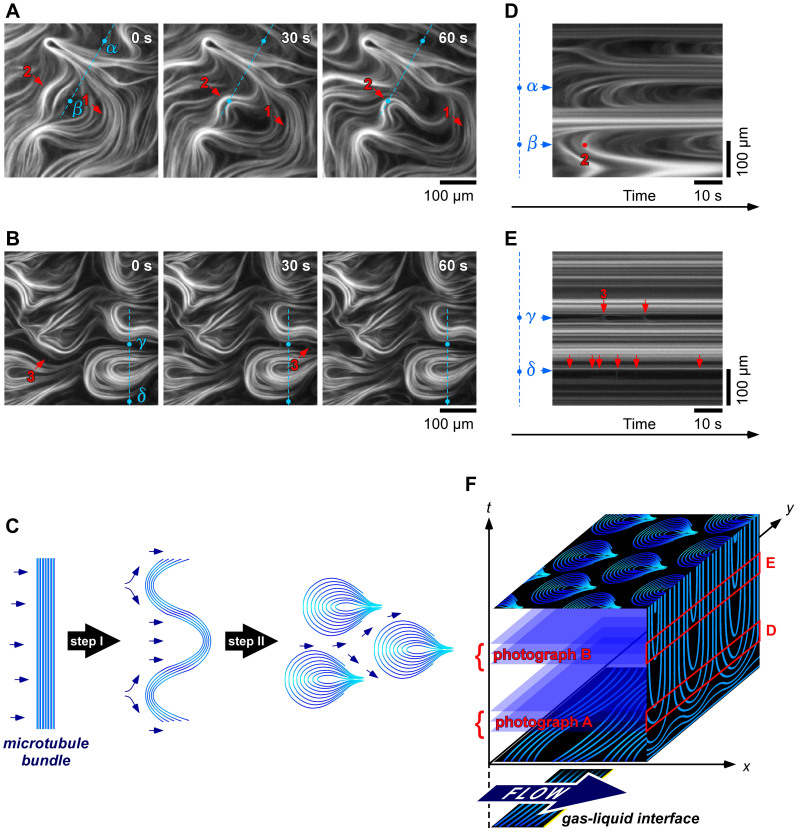
Growth of teardrop patterns, and spatiotemporal analysis. (A), (B), Time courses for formation of microtubule pattern for the first and second steps. Blue dotted line indicates direction perpendicular to hydrodynamic flow. *α*–*δ* represents fixed point on that line. (C), Schematic illustrating formation of microtubule pattern. Microtubule bundle bends under flow force to become nucleus for teardrop pattern (step I). Bundles curve along nucleus or flow through gaps (step II). Arrow indicates flow of microtubule bundles. (D), (E), Spatiotemporal pattern constructed from sequential line-up of dotted lines in A and B. Supporting Movies S1 and S2. (F), Summary of spatiotemporal analysis.

The bundle flow was monitored to clarify the growth process of the patterns (Supporting Movies S1 and S2). The patterns formed in about 20 min. Microtubule bundles in a flow perpendicular to the long axis typically bend through two steps into teardrop patterns. Figure 2A shows the first step of the process (5–6 min. after the samples were set between the glass slides), and Fig. 2B shows the second step (12–13 min. after the samples were set between the glass slides). In the first step, the parallelly oriented bundles integrally flowed to flexibly bend (Fig. 2A, arrow 1). The microtubule bundles inhomogeneously bend under the hydrodynamic flow to form dense layers and more widespread layers (Fig. 2C). The condensed bundles were pinned in an arbitrary position (Fig. 2A, arrow 2) and formed the nucleus as a template for forming the teardrop patterns. In the second step, more bundles subsequently flowed to accumulate on the top of the nucleus. On the other hand, the microtubule bundles in a flow parallel to the long axis formed no patterns (Supporting Information, Fig. S4).

Figures 2D and E show a spatiotemporal diagram constructed from sequential line-up of dotted line in Fig. 2A and B, respectively. The spatial points *α–δ* show the microtubule flowing through the gaps between the nuclei. In the first step, most of the bundles flow with bending and accumulate on the sides of a nucleus, which causes the gaps to narrow (Fig. 2D). In the second step, the bundles flowed through gaps among the nuclei (Fig. 2B, arrow 3). Besides, the gap continuously narrows and the number of flowing bundles decreases to zero (Fig. 2E, and Supporting Information, Fig. S5). The spatiotemporal analysis are summarized in Fig. 2F. The flowed and accumulated bundles were traced on the plane perpendicular to the flow (Fig. 2F, *y*-*t* plane). The temporal fluctuations in the first step were thus resulted in a stable spatial pattern in the second step.

**Figure 3:**
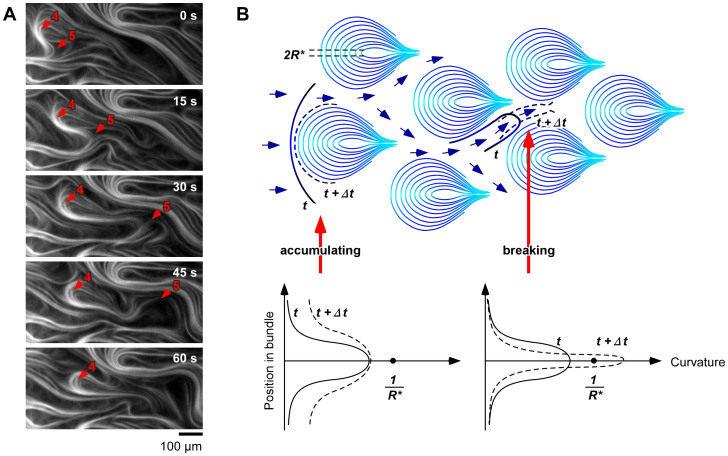
Mechanism of formation of microtubule teardrop pattern. (A), Time courses for bundle accumulation (Arrow 4) and instance of bundle breaking (Arrow 5). See Supporting Movie S1. (B), Schematic illustrating microtubule bundles accumulating along nuclei or breaking as they pass through gaps. Change in curvature of microtubules during accumulation (left) and breaking (right). 1/R* represents critical curvature of microtubules.

The mechanism of the teardrop pattern formation can be described by the flexural rigidity of the microtubules (Fig. 3). When the bundles flowed on the top of the teardrop, the curvature of the bundle tail increased, but it was still lower than the curvature of the teardrop top (Fig. 3A, arrow 4 and Fig. 3B, left). Consequently, the bundles accumulated on the nuclei, and the size of individual teardrop bundles increased. In contrast, when the bundles flowed in the gap between the teardrops, the curvature of the bundle front exceeded the critical curvature of the microtubules, 1/R*, which resulted in the bundles breaking (Fig. 3A, arrow 5 and Fig. 3B, right). Thus, 1/R* should be an important factor for teardrop pattern formation. According to the Bernoulli-Euler curvature and bending moment theory, the curvature (1/*R*) is related to the bending moment as follows:
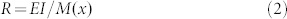


Here, *EI* represents the flexural rigidity and *M*(*x*) represents the bending moment. The bending moment is described by the uniformly distributed load equation:
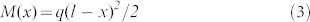


Here, *q* represents the load from the hydrodynamic flow, *l* represents the effective length of a microtubule bundle, and *x* represents the distance from the fixed cantilever point (Supporting Information, Fig. S6)^7^. When the moment reaches a maximum at the top of the teardrop, *i*.*e*., *M*(*0*) = *q1*^2^/2, *R* reaches a minimum value of R*. As shown in Fig. 1B, R* at the top of the teardrop is critical (~1 μm), enabling some bundles to maintain the curved structure while others broke (see Supporting Information, Fig. S7).

**Figure 4:**
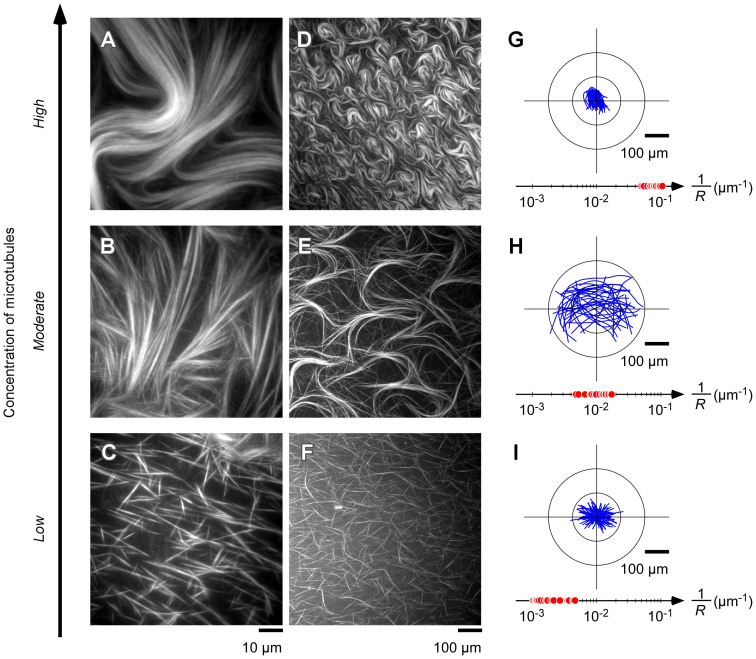
Formation of microtubule pattern at several concentrations. Fluorescence microscope images (A–F). In terms of tubulin concentration: ~400 μM for the high concentration of microtubules (A, D); ~100 μM for the moderate concentration of microtubules (B, E); ~25 μM for the low concentration of microtubules (C, F). Each microtubule bundle shown in images (D–F) is centered at coordinate origin (G–I, blue lines), and *R* represents curvature radius determined from width of teardrop, 2*R* (G–I, red points). Scale bars = 10 μm (A–C); 100 μm (D–I).

To evaluate the effects of the concentration of microtubules on the formation of the patterns and the flexibility of the microtubule bundles, the curvature and effective bending of the microtubule bundles were analyzed. As shown in Fig. 4, teardrop patterns were formed at a high concentration of microtubules. However, at a low concentration, the number of microtubules was insufficient to form bundles, and the microtubules broke into conventional straight pieces (Figs. 4C and F). At a moderate concentration, the microtubules formed bundles, but each one separated at the edge of the bundles (Figs. 4B and E). These results show that the flexibility of the microtubules in a bundle subjected to shear stress causes the bundle to bend plastically along its long axis. Although the bundles at the moderate concentration seemed to be in the process of forming patterns, the edges of the bundles remained in contact and their movement was thus hindered for a long period of time (Supporting Movie S3). As the concentration increased, the bundle curvature 1/*R* increased (Figs. 4G–I). At a high concentration of microtubules, the microtubules in the bundle apparently assisted each other to maintain their positions and reduce the shear strain. To estimate the effective length and the direction of the bundles, each bundle shown in Figs. 4D–F was repositioned at the center axis (Figs. 4G–I). As the concentration increased, the effective bundle length and the number of microtubules in each bundle increased. In addition, the direction from the top to the tail of the teardrop became increasingly oriented along the direction of hydrodynamic flow (Supporting Information, Fig. S8).

In conclusion, teardrop pattern formation of microtubule bundles under a hydrodynamic flow has been discovered. The condensed microtubule bundles in the flow perpendicular to the long axis of the bundles bent to form a nucleus for the pattern on which the subsequent bundles accumulated to grow the pattern. The determining factor in the process appeared to be the critical curvature of microtubules, which depends on their flexural rigidity. The curvature radius of the teardrop nucleus, and that of the bundle breaking point around the gap between the nucleus, were shown to be of critical value. These facts indicate that macromolecules having a high-aspect ratio and an intrinsic flexural rigidity are capable of expressing unique patterns in fluid mechanical energy. Considering that the scale of the teardrop pattern is similar scale of the plant cell wall pattern or multicellular tissue built on microtubules, it could be assumed that physical information such as intrinsic flexural rigidity is an essential factor in determining building blocks in biological organization, as well as chemical information. Such protein pattern formation will provide a simple method of spatial control in novel soft material with hierarchical structures.

## Methods

### Preparation of tubulin

Highly concentrated piperazine-*N*,*N*′-bis(2-ethanesulfonic acid) (PIPES buffer) [1 M PIPES-2K; 20 mM ethyleneglycol-bis(*β*-aminoethyl ether)-*N*,*N*,*N′*,*N′*-tetraacetic acid (EGTA); 10 mM MgCl_2_; KOH used to adjust pH to 6.8] was used to purify tubulin from porcine brain^20^. Microtubule-associated proteins were removed using this protocol. The concentration of tubulin was determined using absorbance at 280 nm with an extinction coefficient of 115,000.

### Preparation of rhodamine-tubulin

Rhodamine-labeled tubulin was prepared using tetramethylrhodamine succinimidyl ester (Invitrogen) according to the method described in a previous study^21^. Rhodamine-tubulin was obtained by chemical crosslinking, and the labeling ratio was 1.2, which was determined by comparing the absorbances of the protein and tetramethylrhodamine molecules at 280 and at 555 nm, respectively.

### Fluorescence microscopy observation of microtubules

Rhodamine-labeled microtubules were obtained by polymerizing rhodamine-tubulin and native tubulin (molar ratio: 0.5:99.5; [tubulin] = ~400 μM) in BRB80 buffer (80 mM PIPES-2K; 5 mM MgCl_2_; 1 mM EGTA; pH 6.8) with 1 mM guanosine-5′-triphosphate (GTP) at 37°C for 15 min. Samples were prepared for a given microtubule concentration by diluting the microtubule solution with BRB80 buffer, including 1 mM GTP. A thin liquid layer of microtubules (~1.5 μL; 18 mm × 18 mm × ~5 μm) was prepared by placing a cover glass (18 mm × 18 mm) on a sample droplet on a slide glass (see Supporting Information Fig. S1). The microtubules were observed using a fluorescence microscope (IX71, Olympus) with objective lenses (Olympus, UPLSAPO 10X/0.40; Nikon, Plan Apo 100X Oil/1.45) and an electron-multiplying charge-coupled device (EM-CCD) camera (Andor iXon+, AndorTechnology plc., Belfast, Northern Ireland) at room temperature (~25°C).

## Author Contributions

K.O. designed and conducted the experiments, and wrote the paper. R.K. and R.Y. contributed to the interpretation. Y.O. supervised the research and edited the manuscript.

## Supplementary Material

Supplementary Information Supporting Information

Supplementary Information Movie S1

Supplementary Information Movie S2

Supplementary Information Movie S3

## References

[b1] Nedelec F., Surrey T., Maggs A. C. & Lebler S. Self-organization of microtubules and motors. Nature 389, 305 (1997). 9305848 10.1038/38532

[b2] Kawamura R., Kakugo A., Shikinaka K., Osada Y. & Gong J. P. Ring-shaped assembly of microtubules shows preferential counterclockwise motion. Biomacromolecules 9, 2277 (2008). 18662029 10.1021/bm800639w

[b3] Sumino Y. *et al.* Large-scale vortex lattice emerging from collectively moving microtubules. Nature 483, 448 (2012). 22437613 10.1038/nature10874

[b4] Sanchez T. *et al.* Spontaneous motion in hierarchically assembled active matter. Nature 491, 431 (2012). 23135402 10.1038/nature11591 PMC3499644

[b5] Tolomeo J. & Holley M. C. Mechanics of microtubule bundles in pillar cells from the inner ear. Biophys. J. 73, 2241 (1997). 9336220 10.1016/S0006-3495(97)78255-9 PMC1181125

[b6] Heussinger C., Bathe M. & Frey E. Statistical mechanics of semiflexible bundles of wormlike polymer chains. Phys. Rev. Lett. 99, 048101 (2007). 17678408 10.1103/PhysRevLett.99.048101

[b7] Howard J. Mechanics of motor proteins and the cytoskeleton. Snauer Associates, Inc. (2001).

[b8] Pampaloni F. *et al.* Thermal fluctuations of grafted microtubules provide evidence of a length-dependent persistence length. Proc. Natl. Acad. Sci. USA 103, 10248 (2006). 16801537 10.1073/pnas.0603931103 PMC1502443

[b9] Venier P., Maggs A. C., Carlier M. F. & Pantaloni D. Analysis of microtubule rigidity using hydrodynamic flow and thermal fluctuation. J. Biol. Chem. 269, 13353 (1994). 7909808

[b10] Tabony J. Morphological bifurcations involving reaction-diffusion processes during microtubule formation. Science 264, 245 (1994). 8146654 10.1126/science.8146654

[b11] Needleman D. *et al.* Higher-order assembly of microtubules by counterions. From hexagonal bundles to living necklaces. Proc. Natl. Acad. Soc. USA 101, 16099 (2004). 10.1073/pnas.0406076101 PMC528963 15534220

[b12] Liu Y., Guo Y., Valles J. M. Jr & Tang J. X. Microtubule bundling and nested buckling drive stripe formation in polymerizing tubulin solutions. Proc. Natl. Acad. Sci. USA 103, 10654 (2006). 16818889 10.1073/pnas.0510381103 PMC1502287

[b13] Karsenti E., Nédélec F. & Surrey T. . Modelling microtubule pattern. Nat. Cell Biol. 8, 1204 (2006). 17060901 10.1038/ncb1498

[b14] Kakugo A. *et al.* Formation of well-oriented microtubules with preferential polarity in a confined space under a temperature gradient. J. Am. Chem. Soc. 131, 18089 (2009). 19928896 10.1021/ja901538n

[b15] Sanchez T., Welch D., Nicastro D. & Dogic Z. Cilia-like beating of active microtubule bundles. Science 333, 456 (2011). 21778400 10.1126/science.1203963 PMC3172966

[b16] Liu L., Tüzel E. & Ross J. L. Loop formation of microtubules during gliding at high density. J. Phys. Condens. Matter. 23, 374 (2011). 10.1088/0953-8984/23/37/374104 21862840

[b17] Brokaw C. J. Direct measurements of sliding between outer doublet microtubules in swimming sperm flagella. Science 243, 1593 (1989). 2928796 10.1126/science.2928796

[b18] Lenaghan S. C. *et al.* High speed microscopic imaging of flagella motility and swimming in giardia lamblia trophoziotes. Proc. Natl. Acad. Sci. USA 108, E550 (2011). 21808023 10.1073/pnas.1106904108 PMC3161553

[b19] Iida J. *et al.* The projection domain of MAP4 suppresses the microtubule-bundling activity of the microtubule binding domain. J. Mol. Biol. 320, 97 (2002). 12079337 10.1016/S0022-2836(02)00402-3

[b20] Castoldi M. & Popov A. V. Purification of brain tubulin through two cycles of polymerization-depolymerization in a high-molarity buffer. Protein Expression Purif. 32, 83 (2003). 10.1016/S1046-5928(03)00218-3 14680943

[b21] Peloquin J., Komarova Y. & Borisy G. Conjugation of fluophores to tubulin. Nat. Methods 2, 299 (2005). 16167385 10.1038/nmeth0405-299

