# Supplementary material for: Microtubule teardrop patterns
Source: Sci Rep. 2015 Mar 31;5:9581. doi: 10.1038/srep09581 (PMC4379470; doi:10.1038/srep09581)

Supporting Information

Microtubule Teardrop Patterns

Kosuke Okeyoshi[1], Ryuzo Kawamura[1], Ryo Yoshida[2], and Yoshihito Osada[1]*

[1]RIKEN Advanced Science Institute, 2-1 Hirosawa, Wako-shi, Saitama 351-0198, Japan.

[2]Department of Materials Engineering, Graduate School of Engineering,

The University of Tokyo, 7-3-1 Hongo, Bunkyo-ku, Tokyo 113-8656, Japan.

Phone: +81 48 467 2816, Fax: +81 48 467 9300

*osadayoshi@riken.jp

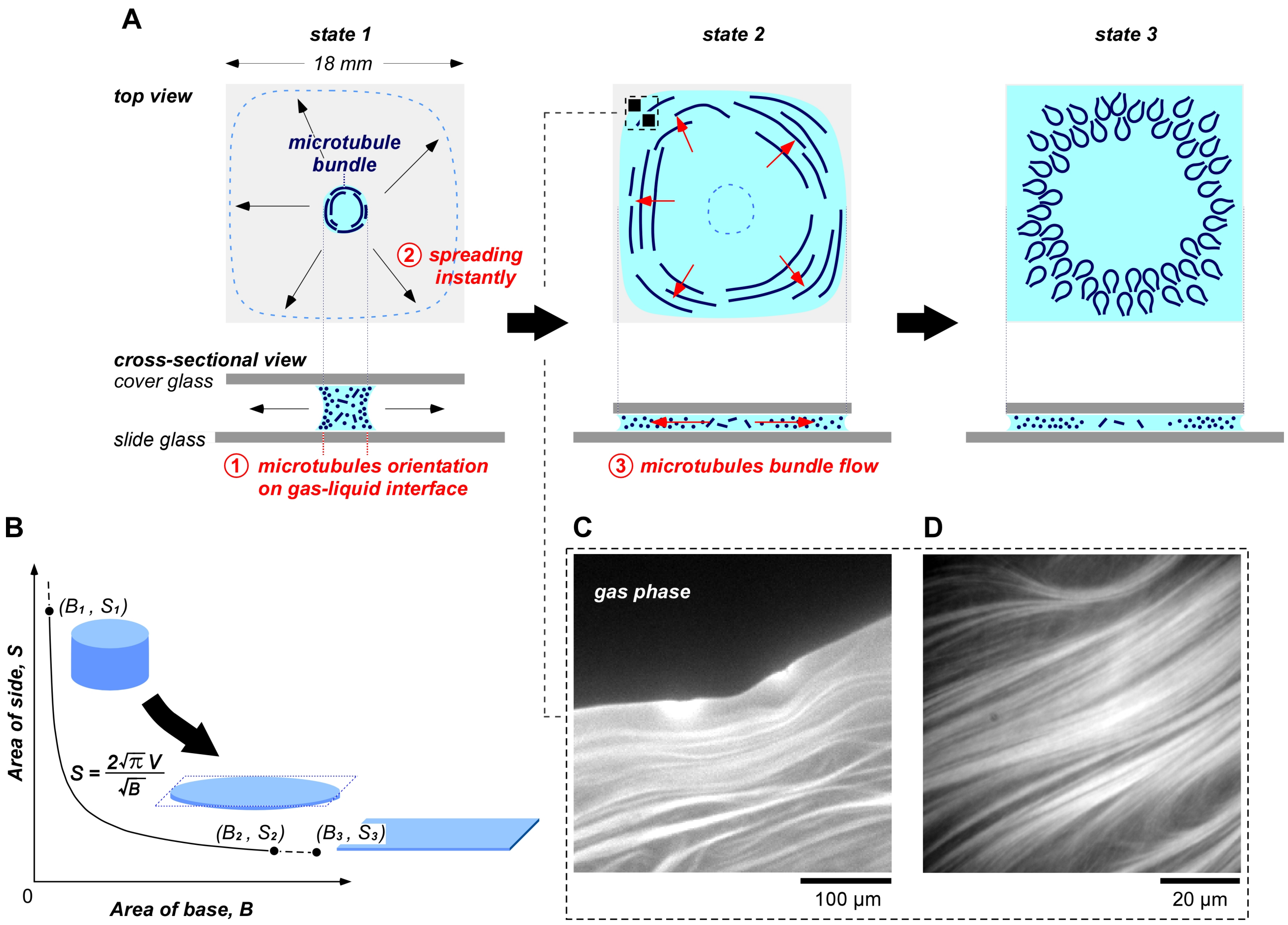

Fig. S1. Preparation of samples of microtubule bundles on gas-liquid interface. **A**, Schematic illustrating microtubule bundle formation from top and cross-sectional views during spreading the solution between glass slides. **B,** Estimation of area of side for a definite volume with cylindrical shape as a function of area of base. B = area of base; S = area of side; V = volume of the solution. **C, D, E,** Fluorescence microscope images. Region around gas-liquid interface (C) and internal liquid phase (D) just after spreading between the glass slides, and internal liquid phase after several tens of minute of the spreading (E). In terms of tubulin concentration: ~400 μM.

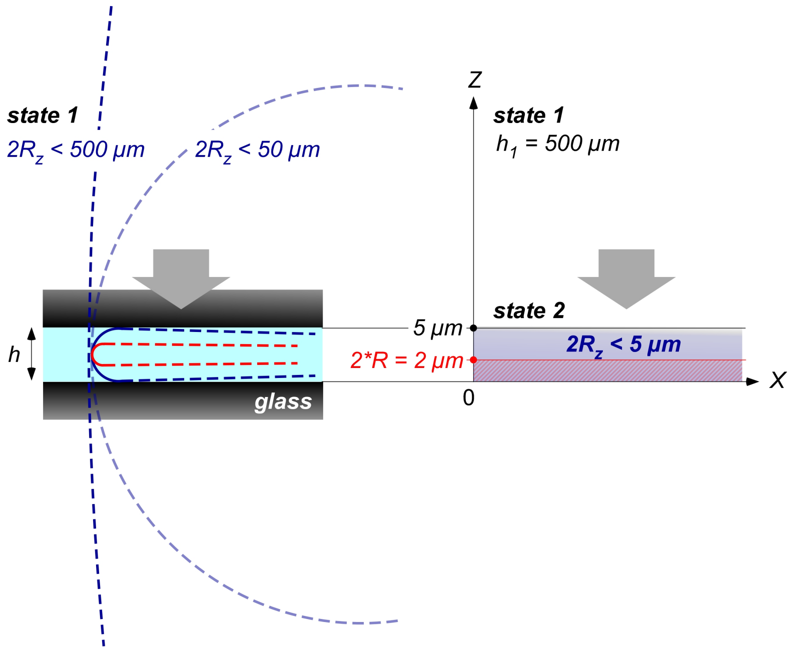

Fig. S2. Microtubule bending limitation in Z-direction between two planes. R_z = bending radius of microtubule in Z-direction.

In the process of the height lowering (**Fig. S1B**), there are three options for the bundles' bending change into the teardrop shape: i) bundle breaking, ii) bending in the Z-axis with a range in the curvature radius (1 μm $< R_Z <$ 2.5 μm), and iii) bending in the direction of the XY-plane. Considering this limitation, the microtubule bundles need to bend with a curvature radius less than the liquid layer height to form a teardrop shape. This limitation for shape change in the Z-direction ($2R_Z <$ 5 μm, **Fig. S2**) causes the bending mainly in the XY-plane. Actually, the teardrop shape is observed in the XY-plane with larger $2R$ (~100 μm) (**Fig. 1B and 1C**).

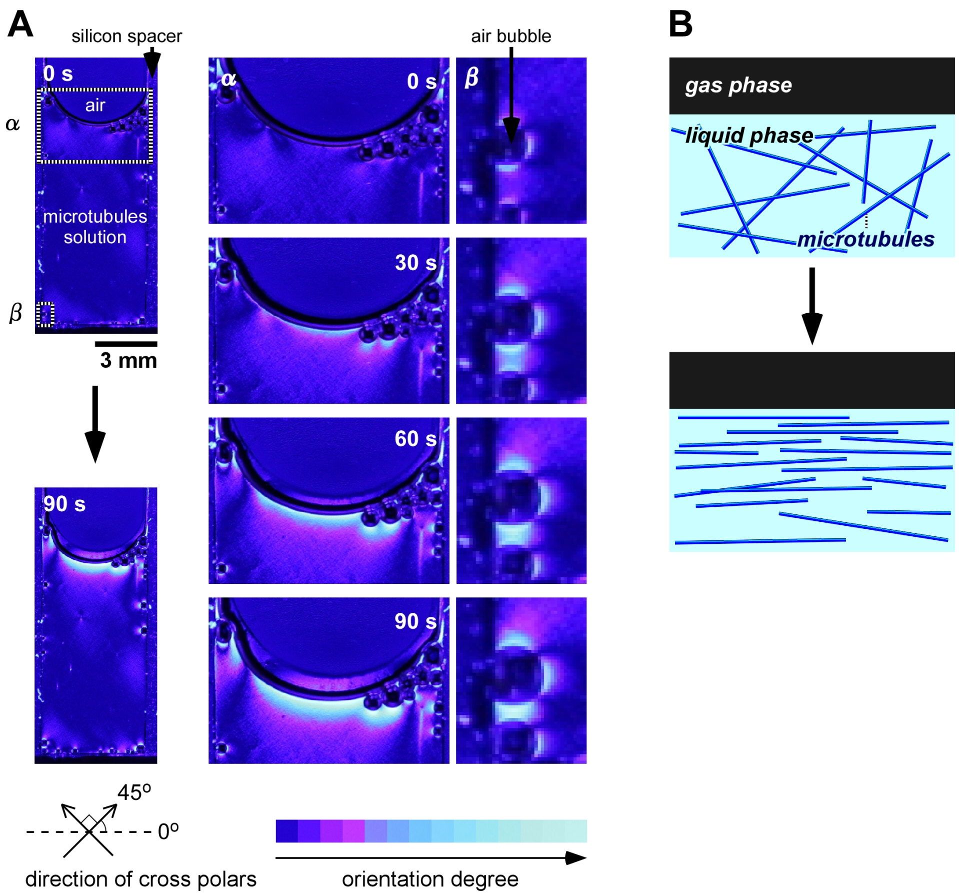

Fig. S3. Microtubules orientation on gas-liquid interface. **A.** Cross polars light observation of microtubules under constant temperature (~26°C). The parts of α and β show the gas-liquid interfaces. In terms of tubulin concentration: ~100 μM. Thickness of the microtubules solution: 500 μm. **B.** Schematic illustration of microtubules orientation on gas-liquid interface.

To verify microtubule bundle formation on a gas-liquid interface, the microtubules solution in a cell using two glass slides and a silicon spacer was observed through cross-polarizers (**Fig. S3A**). Estimating that the solution sample (~1.5 μL) at *state 1* in Fig. S1B is a cylindrical shape with a circular base of 2 mm diameter, h_1 is calculated to be ~480 μm. Therefore, samples were prepared with 500 μm thickness. While the inside of the liquid phase and the interface of liquid-silicon show no color change, the gas-liquid interface shows remarkable color change from dark blue to bright aqua blue. Furthermore, the bright aqua blue part grows from the gas-liquid interface. Observing through cross-polarizers (45°, 135°), the most remarkable color change is in horizontal or vertical directions. The color change in the region β clearly shows the microtubules' orientation on gas-liquid interface around a circular air bubble. Notably, the color change is only in 0° or ±90° directions, but not in ±45° directions. From these results, it is revealed that randomly-existed microtubules form the bundle, and show parallel orientation on the gas-liquid interface (**Fig. S3B**).

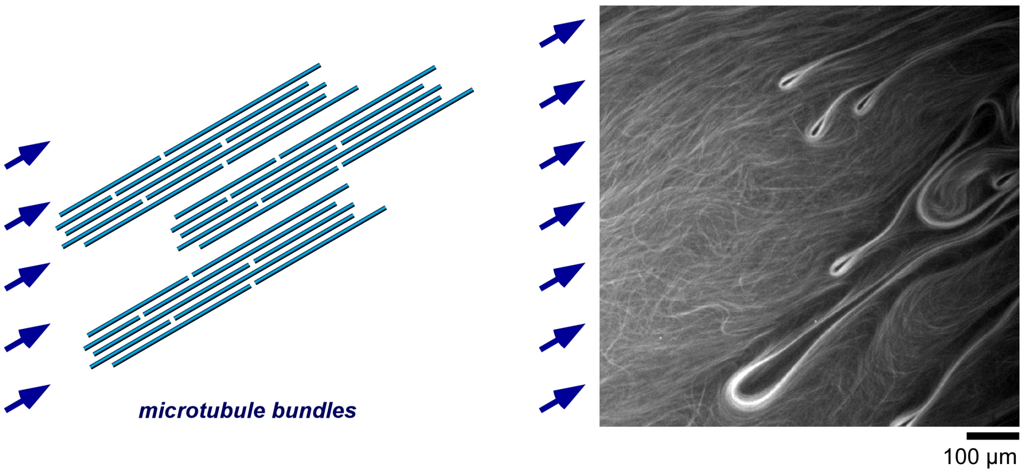

Fig. S4. Microtubule bundles in flow parallel to long axis. Arrows indicate direction of hydrodynamic flow.

When the hydrodynamic flow was parallel to the long axis of the microtubule bundles, most of the bundles deformed into separate microtubules and randomly bent. The top portion of the image shows parallel bundles oriented along the direction of the hydrodynamic flow. These bundles also gradually deformed into separate microtubules. Moreover, some nuclei of long-tailed teardrop patterns were occasionally observed. The teardrop patterns did not grow and the long tails remained because the bundles perpendicular to the hydrodynamic flow and the accumulating bundles were small.

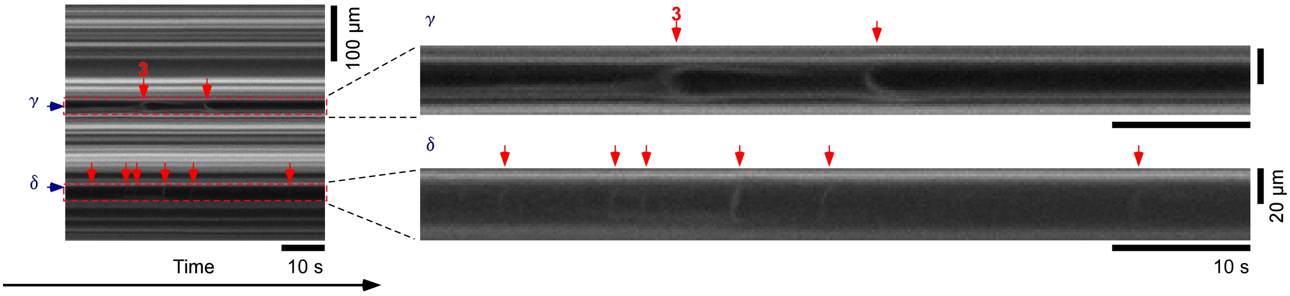

Fig. S5. Spatiotemporal pattern formed during second step (Fig. 2E).

Large images show clear flow of microtubule bundles on gaps shown in Fig. 2E. Bundles could flow through gap until width of gap reached 2 × R^*. See Supporting **Movie S2**.

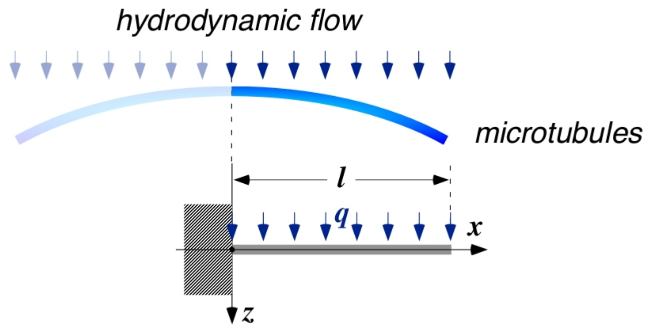

Fig. S6. Schematic of cantilever model illustrating microtubule bundle bending under hydrodynamic flow. q = load from hydrodynamic flow; l = effective length of microtubule bundle; x = distance from fixed cantilever point.

The bending properties were analyzed using cantilever model.[1] Top of teardrop patterns was considered as clamped point.

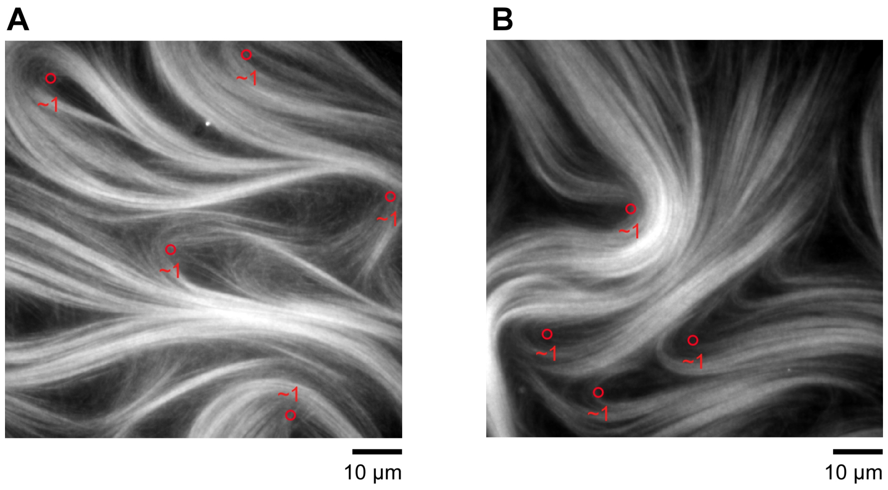

Fig. S7. Fluorescence microscopy images of microtubules for estimating minimum curvature radius, R^*. **A** = Fig. 1B; **B** = Fig. 4D.

Radius of inner core, R, was measured as ~1 μm. On basis of depth of layer (~5 μm), minimum curvature radius, R^*, should range from 1 to 5 μm.

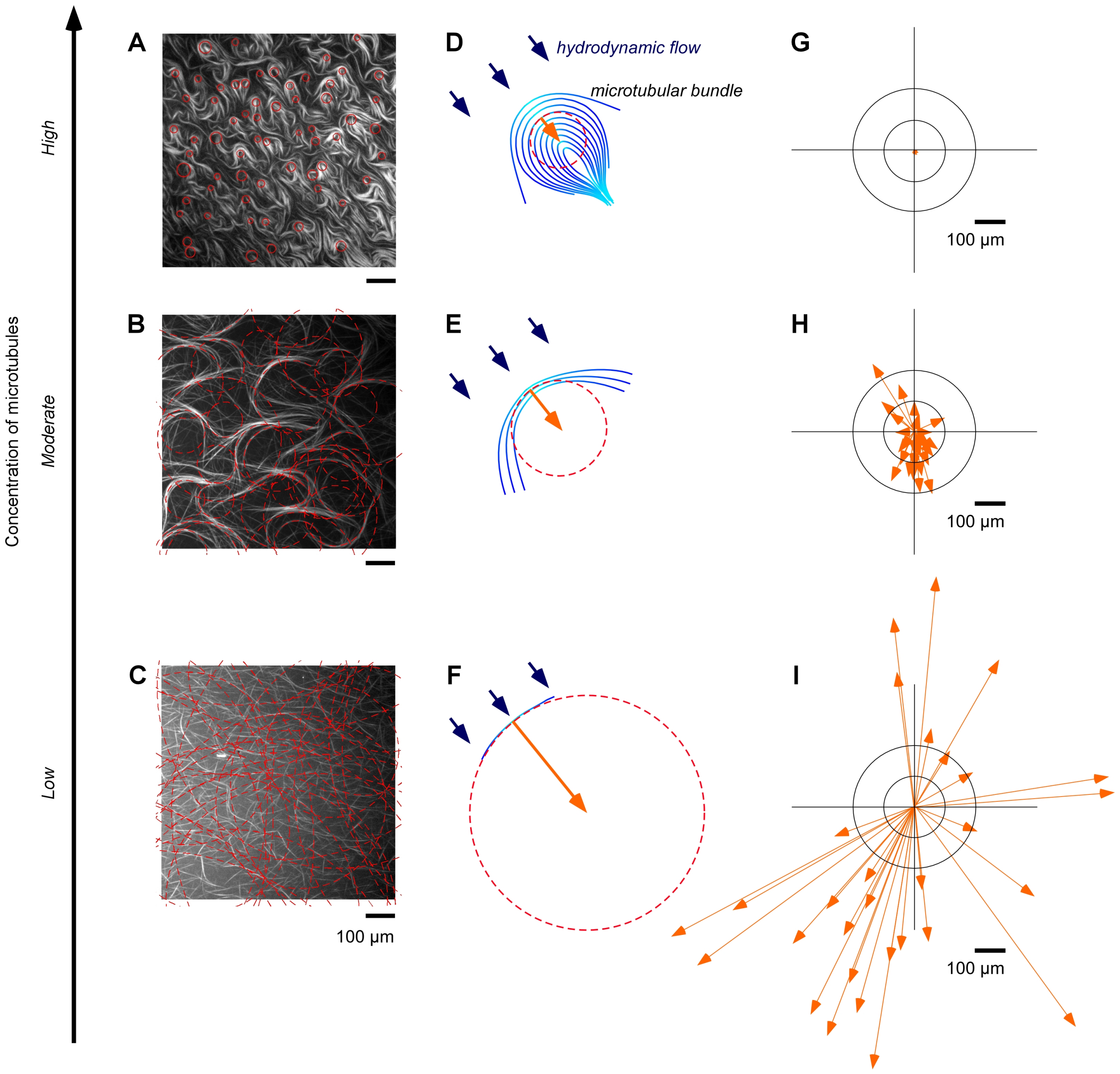

Fig. S8. Effect of concentration of microtubules in hydrodynamic flow on bending properties of microtubule bundles. **A, B, C,** Trace of microtubule bundles in the middle by circular arc (red dotted arc) to analyse the curvatures, $1/R$. **D, E, F,** Circular arc along middle of bundles, and vector from top of curved bundles to center of circle. **G, H, I,** Each vector centered at starting point. Tubulin concentrations: ~400 μM for high concentration of microtubules (**A, D, G**); ~100 μM for moderate concentration of microtubules (**B, E, H**); ~25 μM for low concentration of microtubules (**C, F, I**).

A circular arc was traced along the middle of the bundles to compare the effects of the concentration of microtubules on the bending of the microtubule bundles. As shown in **Fig. S8A and G**, most of the vectors are oriented along the direction of the hydrodynamic flow, and most of the radius of the circle is smaller than 10 μm at the high concentration of microtubules. These results conclude that the direction of hydrodynamic flow and the flexural rigidity of microtubules are determining factors for fabrication of the teardrop patterns. As shown in **Fig. S8H**, some vectors are oriented along the direction of the hydrodynamic flow and others are oriented in different directions at the medium concentration of microtubules. Moreover, the vector length ranged from 50 to 250 μm, which means that the bundles were in the process of pattern formation. However, the microtubule bundles could not be denser because no subsequent microtubule bundles accumulated. As shown in **Fig. S8I**, the vectors show random directions at the low concentration of microtubules. Statistically, many vectors are perpendicular to the hydrodynamic flow because the bundles separated into individual microtubules and formed conventional straight pieces whose long axis easily followed the direction of the hydrodynamic flow.

Movie S1-S2

From the **Movie S1** and **Movie S2**, we can observe some microtubules fixed on the glass surface. This suggests that there is an atmosphere for contact between the microtubules fixed on the glass and free flowing microtubules. Furthermore, there are some microtubule bundles which spontaneously form the nuclei and flow (**Fig. 2A and 2D, arrow 2**). Due to this, Fig. 2C is a basic mechanism even if there are some effects of the roughness of the glass slides' surface.

References

1. Howard, J. Mechanics of motor proteins and the cytoskeleton. Ch. 6, 99-111 (*Suauer Associate, Inc.* 2001).

Supporting Movie Captions

Movie S1. Microtubule bundle pattern formed during first step (5–6 min).

Movie S2. Microtubule bundle pattern formed during second step (12–13 min).

Movie S3. Microtubule bundles formed at moderate concentration. [Tubulin] = ~100 μM.

800-μm square field at 10 times actual speed was used for all movies.

Movie S1

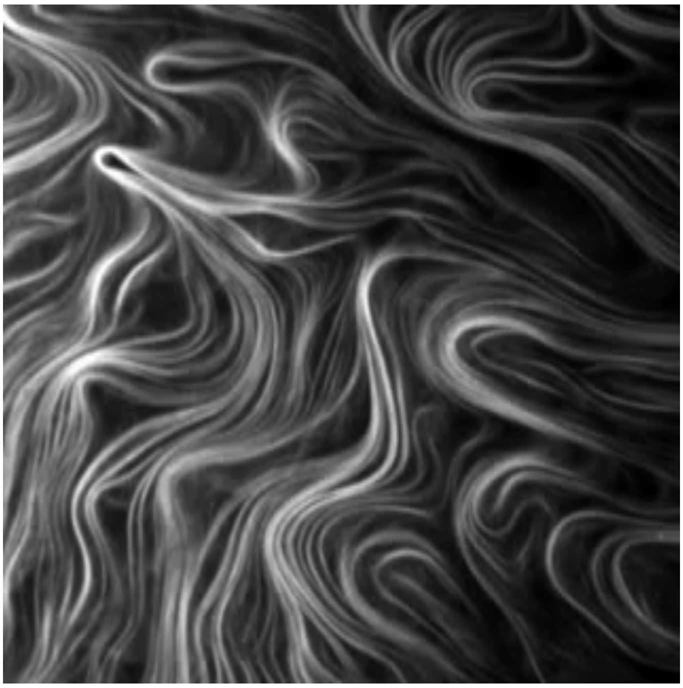

Movie S2

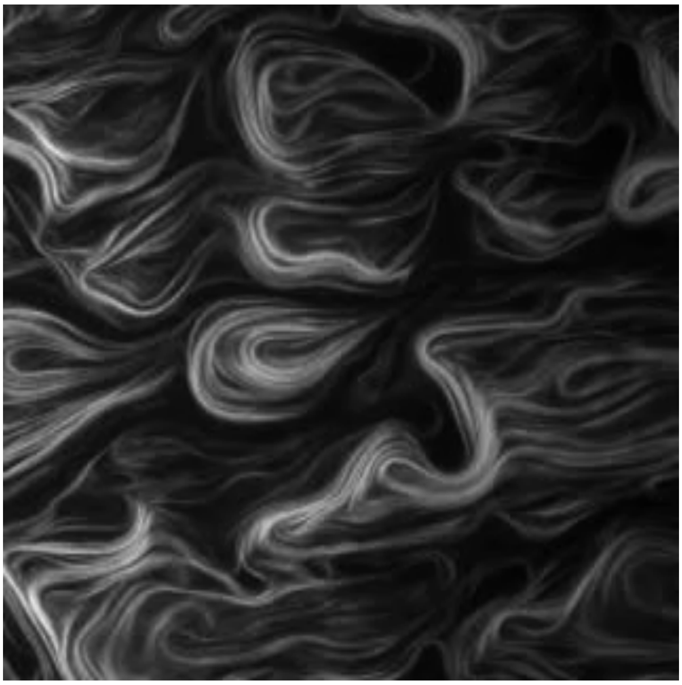

Movie S3

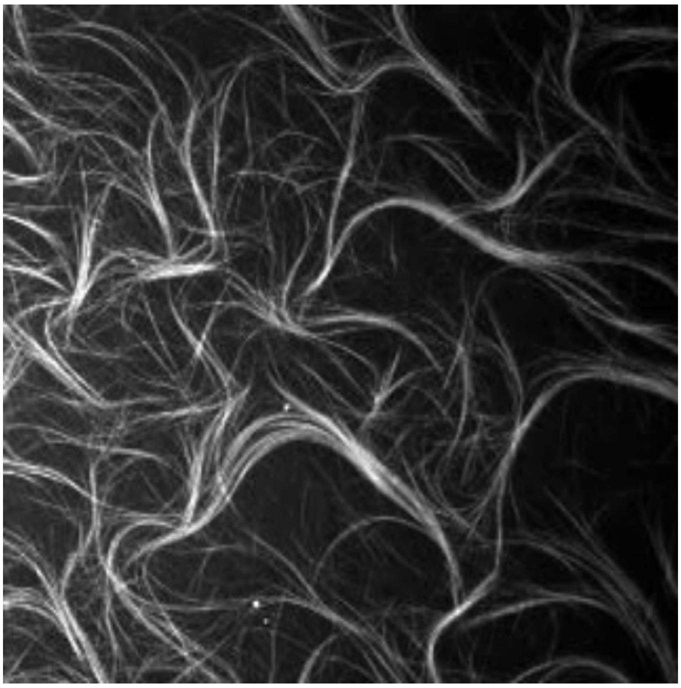

Supplement: Supplementary Information — Supporting Information [file srep09581-s1.pdf]
